# Soil-Transmitted Helminth Infections and Associated Risk Factors among Schoolchildren in Durbete Town, Northwestern Ethiopia

**DOI:** 10.1155/2015/641602

**Published:** 2015-06-16

**Authors:** Tilahun Alelign, Abraham Degarege, Berhanu Erko

**Affiliations:** ^1^Department of Microbial, Cellular and Molecular Biology, College of Natural Sciences, Addis Ababa University, P.O. Box 1176, Addis Ababa, Ethiopia; ^2^Department of Biology, Debre Birhan University, Debre Birhan, Ethiopia; ^3^Aklilu Lemma Institute of Pathobiology, Addis Ababa University, P.O. Box 1176, Addis Ababa, Ethiopia; ^4^Department of Epidemiology, Robert Stempel College of Public Health and Social Work, Florida International University, Miami, FL, USA

## Abstract

Identifying determinants of soil transmitted helminth infection is vital to design control strategy for the disease. This study assessed the prevalence of STH infections and associated factors among schoolchildren in Durbete town, northwestern Ethiopia. Data about the sociodemographic and socioeconomic status of the children were collected using a questionnaire and stool samples were diagnosed using thick Kato-Katz smear. STH infection was more common among school-age children in Durbete town. Hookworm was the most frequent helminth species detected. The prevalence of STH infection was more in children who did not practice wearing shoes and washing hands before eating and in those who were older in age. Deworming of school-age children in the study area would be important. In addition, provision of health education about helminths and the importance of wearing shoes and washing hands before eating would be important to reduce the burden of STH infection in the study area.

## 1. Background

Infection with soil-transmitted helminths (STHs) is common in the world particularly in the rural developing regions. About 2 billion people in the world are infected with at least one species of STH (due to* Ascaris lumbricoides,* 1 billion,* Trichuris trichiura,* 800 million, and hookworm, 740 million) and 4 billion are at risk of infection [1]. STH infections are especially prevalent among children, negatively affecting their growth, intellectual development, and resistance to other diseases [2]. More than 613 million school-age children in the world are at risk of STH infection [3].

Warm climates and adequate moisture, lack of personal or environmental hygiene, sanitation, and education, walking barefoot, and poor health or nutritional status could increase the risk of STH infections [4–7]. Due to their frequent playing habits and low level of awareness, STH infection is highly prevalent among school-age children [8–10].

In Ethiopia, STH infection is a serious public health problem [8–10]. The prevalence of STH infection reaches up to 83.3% in some regions of the country [11]. Risk factors associated with STH infections could vary with localities and thus such information is vital to guide policy makers in designing a more focused preventive approach to control the disease. Therefore, the present study was undertaken to determine the prevalence of STH infections and their associated determinants among schoolchildren in Durbete town, northwestern Ethiopia.

## 2. Material and Methods

### 2.1. Study Area and Population

The study was conducted among 384 children in Abchikeli (247) and Ayalew Mekonnen (137) Elementary Schools located in Durbete town, northwestern Ethiopia, in February and March 2010. Worm infections are prevalent among communities living in the town (based on information obtained from the town health center). The town lies at 1500 meters above sea level. The area has annual temperature and rain fall range of 25°C to 29°C and 1,400 mm to 1,594 mm, respectively.

### 2.2. Sample Size Estimation

The sample size for the study was calculated using the formula *n* = (*zα*/2)^2^ *p*(1 − *p*)/*d* ^2^ at 95% confidence interval (CI) (*zα*/2 = 1.96), 5% margin of error, and 5% nonresponse rates. The prevalence of undernutrition and/or anaemia is not known among school-age children in Durbete town. Thus, the prevalence was assumed to be 50% and sample size was estimated to be 403. The 403 sample was then proportionally divided among Abchikeli (259) and Ayalew Mekonnen (143) Elementary Schools. The sample size determined was then proportionally divided by all grade levels from 1 to 6 in each school and study participants were then selected from each section on a random basis.

### 2.3. Stool Examination

Stool samples collected from children between 10:00 a.m. and 4:00 p.m. were processed using single Kato-Katz thick smear and microscopically examined for ova of intestinal helminths in the field [12]. Eggs were counted within 1 hour of Kato smear preparation for hookworms and later within a week for* A. lumbricoides *and* T. trichiura *at the Aklilu Lemma Institute of Pathobiology. Then, the intensity of infection was estimated according to the WHO guidelines [7].

### 2.4. Sociodemographic and Socioeconomic Factors

A pretested standardized questionnaire was developed in English and translated into Amharic before interviewing. Children were then asked for information about socioeconomic and sociodemographic characteristics.

### 2.5. Data Analysis

Data were entered and verified using Microsoft Excel 2007 and analysis was performed using Stata version 11 (Stata Corporation, College Station, Texas, USA). *Z*-test was used to test the difference in the prevalence of intestinal helminth infection by age and sex groups, school of children, and place of residence. Logistic regression analysis was used to measure the strength of association between different socioeconomic and sociodemographic explanatory factors with intestinal helminth infections (dependent variable). Values were considered significant when *P* < 0.05.

### 2.6. Ethical Considerations

The study protocol was approved by the Ethical Clearance Committee of the Biology Department, Addis Ababa University (IRB approval number: SF/Biol/1071/02), before its implementation. Health officers of Debub Achefer district, educational authorities, and school principals in the town also granted permission for the study. Stool samples were then collected from children who gave their assent. Parents or guardians of children also gave written informed consent before collecting stool specimens. All children who had intestinal helminth infections were treated with appropriate dose of mebendazole.

## 3. Results

### 3.1. Prevalence of Intestinal Helminth Infections

About 54.9% (211) of children examined were infected with at least one intestinal helminth species. Hookworms,* A. lumbricoides*,* T. trichiura*,* E. vermicularis*, and* H. nana* infections were observed in 46.9%, 13.9%, 2.3%, 1.1%, and 0.5% of the children, respectively (Table 1). Prevalence of intestinal helminth infection was significantly higher in children of ages 10 to 14 years than children of ages 5 to 9 years (*P* < 0.01) and in children enrolled at Abchikeli Elementary School than those enrolled at Aylew Mekonnen Elementary School (*P* < 0.01). The difference in the prevalence of intestinal helminth infection was also significant between children living in rural and urban regions (*P* < 0.01). Similarly, hookworm infection was more common in children of ages 10 to 14 years and enrolled at Abchikeli Elementary School than children of ages 5 to 9 years and enrolled at Aylew Mekonnen Elementary School, respectively (*P* < 0.01). The prevalence of hookworm infection was higher among children living in rural areas than children living in urban areas (*P* < 0.01). However, the prevalence of* A. lumbricoides*,* T. trichiura*,* E. vermicularis*, and* H. nana* infections was similar in children enrolled at the two schools or between children in the ages 5 to 9 and 10 to 14 years. Prevalence of intestinal helminth infection was comparable in males and females. The prevalence of single, double, and triple infections was 45.8%, 8.6%, and 0.5%, respectively.

Out of 180 children found positive for hookworm infection, 93.9%, 3.9%, and 2.2% had light (1–1,999 eggs per gram (EPG)), moderate (2,000–3,999 EPG), and heavy (≥4,000 EPG) intensity of infection, respectively. However, all the children infected with* A. lumbricoides* had light intensity (1–4999 EPG) of infection with the parasite and 88.9% (8/9) of the children infected with* T. trichiura* had light intensity of* Trichuris* infection (1–999 EPG).

### 3.2. Association of Intestinal Helminth Infection with Socioeconomic and Sociodemographic Factors

The odds of STH infections were significantly higher in children of ages 10 to 14 years than in children of ages 5 to 9 years [adjusted odds ratio (AOR) = 2.79, 95% CI (confidence interval) = 1.56, 5.01]. Children who did not wear shoes [AOR = 2.42, 95% CI = 1.05, 5.57] and did not have the habit of washing hands before eating [AOR = 3.80, 95% CI = 1.02, 14.23] had higher chance of being infected with STH than children who wear shoes and had the habit of washing their hands before eating, respectively. The difference in the odds of STH infection between children of ages 5 to 9 years and 10 to 14 years [AOR = 4.66, 95% CI = 1.29, 16.75] or between children who wear shoes and those who do not wear shoes [AOR = 6.03, 95% CI = 1.58, 22.95] was particularly high in the case of hookworm infection.

The likelihood of STH infection in children who used tap water for drinking and latrine for defecating was comparable with the chance of infection with the parasite among children who drink river, spring, or well water and those who did not have latrine, respectively (Table 2). The odds of intestinal helminth infection were also similar between children who had literate and illiterate family and between children who lived in cement and earthen floor house. The odds of intestinal helminth infection were also similar in males and females.

## 4. Discussions

The overall prevalence of STH infection observed among schoolchildren in the current study (54.9%) was relatively high. The majority of the study participants did not use latrine (60.7%), did not use tap water for drinking (66%), did not wear shoes (77.6%), lived in a rural area (64.3%) in houses with earthen floor (94.5%), and had illiterate mothers (62.8%) and fathers (52.6%). These factors could contribute to the high prevalence of STH observed among schoolchildren living in the study area. Most reports in Sub-Saharan Africa regions including Ethiopia documented a higher prevalence of* A. lumbricoides* and* T. trichiura* infection followed by hookworm [13, 14]. Globally, the highest number of estimates of STH infections was also attributed to* A. lumbricoides* and* T. trichiura* infection followed by hookworm [15]. However, the prevalence of hookworm infection in the current study was significantly higher than the prevalence of* A. lumbricoides* and* T. trichiura* infections and the prevalence of the parasite estimated for the country (16%) [13]. This finding is unexpected as the study participants were schoolchildren. The prevalence of hookworm is high particularly in adults [16]. However, the observed high prevalence of hookworm infection among children studying in Abchikeli Elementary School in Durbete town could be due to the fact that most children studying in elementary schools in the town did not wear shoes and they played or walked over loamy soils and cultivated fields.

As expected and also previously reported [5, 17], habits of not wearing shoes and not washing hands before eating were associated with increased odds of STH infection. Hookworm infection occurs due to penetration of the skin by the larvae of the parasite. As a result, children walking barefoot on the soil contaminated with fecal matter will be exposed to the infective larval stages of the parasite. Similarly, children playing in contaminated soil might also get exposed to infective stages (embryonated eggs) of* A. lumbricoides*.

The odds of STH infection were also greater among children of ages 10 to 14 years than those of lower age groups. A similar previous study also documented a higher prevalence of STH infection among children of ages 10 to 14 years than children of ages 5 to 9 years [18]. Children of ages 5 to 9 years are usually under close care of their parents and will be more protected from infection than older age children. On the other hand, children of ages 10 to 14 years are physically strong and as a result usually play in open fields and in fecal contaminated soil, exposing themselves to STH infection. In addition, the odds of STH infection among children in the 10-to-14-age group were higher than those in the 5-to-9-age group. This result could be due to the predominance of hookworm (rather than* A. lumbricoides* and* T. trichiura*) infection among the current study participants. Prevalence and intensity of* A. lumbricoides* infection rose to the peak in the 5-to-10-age group and declined thereafter [19]. However, prevalence of hookworm infection rose with age until the 15-to-20-year age group [19]. Similarly, the prevalence of hookworm infection in the current study increased with an increase in the age of children. Hookworm tends to be more of an occupational infection, and older children might catch the infection while helping their parents with agricultural activities in the field.

The prevalence of hookworm infection was higher in children living in rural areas than those who live in urban areas. However,* A. lumbricoides* and* T. trichiura* infections were slightly more common among children in urban areas. Another study also reported a similar pattern of prevalence of the aforementioned STH species in rural and urban areas [20]. The prevalence of hookworm infection was also not similar between schools. The prevalence of hookworm infection was higher in children studying at Abchikeli Elementary School than those in Ayalew Mekonnen. This is expected as more than 95% of children studying in Abchikeli Elementary School did not wear shoes and were living in rural areas.

On the other hand, sex of children, education status of families, presence or absence of latrine, house floor nature, and drinking water source were not associated with STH infection among schoolchildren in the current study. This finding is in contrast with some previous reports [7, 17, 21–23]. The topography, ecology, environmental sanitation, life style, and culture of the community may vary among regions. As a result, risk factors for STH infection in one region may not be determinant in other regions.

Only one Kato-Katz slide was examined per stool sample in the present study. As a result, the prevalence of STH infections observed in this study might have been underestimated. In addition, the current study is cross-sectional, making it difficult to make conclusions about the causal relationship between the observed risk factors and STH infections.

In conclusion, STH infection is a major public health problem among schoolchildren in Durbete town, northwestern Ethiopia. The habits of not wearing shoes and washing hands before eating as well as being in the 10-to-14-year age group were associated with increased odds of STH infections among children in the study area. Deworming complemented with other measures such as provision of health education and improvement of sanitation and hygiene maintains reduced level of infection produced by the chemotherapy and eventually contributes to elimination of the helminth infections in the study area.

## Figures and Tables

**Table 1:** Prevalence of soil-transmitted helminth infections among schoolchildren in Abchikeli and Ayalew Mekonnen Elementary Schools, Durbete town, northwestern Ethiopia, February to March 2010.

Variables	Categories	Number examined	Hookworms positive	*A. lumbricoides* positive	*T*. *trichiura* positive	*E*. *vermicularis* positive	*H*. *nana* positive	Any helminth
Age in years	5–9	175	32.57	14.29	2.29	0.57	0.57	43.43
	10–14	209	58.85	13.40	2.39	1.44	0.48	64.59
	*P* value		<0.01	0.80	0.95	0.41	0.90	<0.01

Sex	Male	172	45.35	16.28	2.91	1.16	0.58	55.23
	Female	212	48.11	11.79	1.89	0.94	0.47	54.72
	*X* ^2^ (*P*)		0.3 (0.58)	1.6 (0.21)	0.4 (0.51)	0.1 (0.83)	0.1 (0.88)	0.1 (0.92)

School	Abchikeli	247	62.35	11.74	1.62	1.21	0.81	65.18
	Ayalew Mekonnen	137	18.98	17.52	3.65	0.73	0.00	36.50
	*X* ^2^ (*P*)		66.6 (0.00)	2.47 (0.12)	1.6 (0.21)	0.2 (0.65)	1.12 (0.29)	29.3 (0.00)

Residence	Rural	247	62.35	11.74	1.62	1.21	0.81	65.18
	Urban	137	18.98	17.52	3.65	0.73	0.00	36.50
	*X* ^2^ (*P*)		66.6 (0.00)	2.5 (0.12)	1.6 (0.21)	0.2 (0.65)	1.12 (0.29)	29.3 (0.00)

**Table 2:** Factors associated with soil-transmitted helminth infections among schoolchildren in Abchikeli and Ayalew Mekonnen Elementary Schools, Durbete town, northwestern Ethiopia, February and March 2010.

Variables	Categories	Helminth infection		Crude OR [95% CI]	Adjusted OR [95% CI]
		Yes	No		
Family size	1 to 3	97	110		
	4–6	82	41	2.26 [1.43, 3.60]^*∗*^	1.73 [0.01, 2.95]^*∗*^
	≥7	32	22	1.65 [0.89, 3.02]	0.97 [0.46, 2.06]

Fathers education	Literate	96	86		
	Illiterate	115	87	1.18 [0.79, 1.77]	0.57 [0.25, 1.31]

Mothers education	Literate	68	75		
	Illiterate	143	98	1.61 [1.06, 2.44]^*∗*^	1.31 [0.54, 3.20]

Water sources	Tap	45	86		
	Well	92	56	3.13 [1.92, 5.12]^*∗*^	4.40 [0.76, 25.64]
	River or spring	74	31	4.54 [2.59, 7.99]^*∗*^	6.25 [0.98, 39.77]

Hand washing	Yes	191	170		
habit before eating	No	20	3	5.93 [1.73, 20.31]^*∗*^	3.80 [1.02, 14.23]^*∗*^

Shoe wearing	Yes	24	62		
	No	187	111	4.35 [2.57, 7.36]^*∗*^	2.42 [1.05, 5.57]^*∗*^

Presence of latrine	Yes	54	97		
	No	157	76	3.71 [2.41, 5.71]^*∗*^	4.39 [0.92, 20.94]

House floor	Cement	7	14		
	Earthen	204	159	2.57 [1.01, 6.50]^*∗*^	4.13 [0.09, 195.52]

Residence	Urban	50	87		
	Rural	161	86	3.26 [2.11, 5.03]^*∗*^	0.09 [0.01, 1.37]

Grade	1 to 3	150	129		
	4 to 6	61	44	1.19 [0.76, 1.87]	0.28 [0.13, 0.58]^*∗*^

Religion	Muslim	9	9		
	Christian	202	164	1.23 [0.48, 3.17]	0.41 [0.12, 1.38]

Sex	Male	95	77		
	Female	116	96	0.97 [0.65, 1.46]	0.84 [0.52, 1.34]

Age group	5–9	76	99		
	10–14	135	74	2.37 [1.57, 3.58]^*∗*^	2.79 [1.56, 5.01]^*∗*^

^*∗*^ *P* < 0.05*.*
